# Impact of antibiotics on the human microbiome and consequences for host health

**DOI:** 10.1002/mbo3.1260

**Published:** 2022-01-13

**Authors:** Dhrati V. Patangia, Cornelius Anthony Ryan, Eugene Dempsey, Reynolds Paul Ross, Catherine Stanton

**Affiliations:** ^1^ School of Microbiology University College Cork Cork Ireland; ^2^ Teagasc Food Research Centre, Moorepark Fermoy Co. Cork Ireland; ^3^ APC Microbiome Cork Ireland

**Keywords:** antibiotic resistance, gut bacteria, microbiome

## Abstract

It is well established that the gut microbiota plays an important role in host health and is perturbed by several factors including antibiotics. Antibiotic‐induced changes in microbial composition can have a negative impact on host health including reduced microbial diversity, changes in functional attributes of the microbiota, formation, and selection of antibiotic‐resistant strains making hosts more susceptible to infection with pathogens such as *Clostridioides difficile*. Antibiotic resistance is a global crisis and the increased use of antibiotics over time warrants investigation into its effects on microbiota and health. In this review, we discuss the adverse effects of antibiotics on the gut microbiota and thus host health, and suggest alternative approaches to antibiotic use.

## Graphical abstract

Antibiotics can have several negative impacts on host health; both direct and indirect effects. The gut microbiota plays a crucial role in host health regulation. Effects of antibiotics on the host through the gut microbiome are immense and can affect various functions including immune regulation, metabolic activities, and thus overall health.

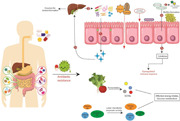

## 1 INTRODUCTION

Since their discovery, antibiotics have revolutionized the treatment of infectious diseases on a global scale. They are recognized as one of the contributing factors to increased life expectancy in the 20th century owing to the decline in infectious disease mortality (Adedeji, 2016). However, their overuse and misuse in human and veterinary medicine and animal husbandry have resulted in the current global antibiotic resistance crisis (Llor & Bjerrum, 2014; Vidovic & Vidovic, 2020) which is exacerbated by the slow rate of new drug development (Simpkin et al., 2017). Despite this, antibiotics are still widely prescribed in disease treatment and studies have reported increased consumption of antibiotics in certain countries in the past number of years (Adriaenssens et al., 2011; Klein et al., 2018).

More recently, scientists have uncovered the detrimental impact of broad‐spectrum antibiotics on the gut microbiota. Home to bacteria, archaea, microeukaryotes, and viruses, the gut microbiota plays a fundamental role in human health. It prevents pathogen colonization, regulates gut immunity, provides essential nutrients and bioactive metabolites, and is involved in energy homeostasis (Mills et al., 2019). In infants, the gut microbiota is acquired during birth and thereafter plays an essential role in the development of infant gut immunity. Evidence to date strongly suggests that balanced microbiota composition and rich species diversity are essential to its optimal functioning (Heiman & Greenway, 2016), which can be compromised in disease states (Mosca et al., 2016). Likewise, reduced diversity and imbalanced microbiota composition in the infant's gut are associated with intestinal illnesses and a predisposition to certain diseases later in life (Milani et al., 2017; Volkova et al., 2021).

Broad‐spectrum antibiotics reduce gut microbiota diversity (Dubourg et al., 2014), and as well as killing the pathogen of concern can eradicate beneficial microbes (Blaser, 2011), with deleterious consequences for the host. Despite this, in Western countries, up to 35% of women are exposed to an antibiotic during pregnancy and delivery, and antibiotics comprise 80% of the drugs a woman is exposed to during pregnancy (Kuperman & Koren, 2016; Stokholm et al., 2013). Mothers are frequently prescribed intrapartum antibiotics prophylactically to prevent and treat infections (Verani et al., 2010).

*Clostridioides difficile* (formerly known as *Clostridium difficile*) infection is an example of a disease brought about directly through antibiotic disruption of the gut microbiota (Theriot et al., 2014). Illness ranges from mild diarrhea to death (Guh & Kutty, 2018). Antibiotic eradication of beneficial bacteria in the gut enables *C. difficile* to flourish (Rea et al., 2011). A recent study also concluded that oral antibiotic use is associated with an increased risk of colon cancer (S. Zhang & Chen, 2019). These are just some of the examples of how antibiotic therapy can compromise health.

**Figure 1:**
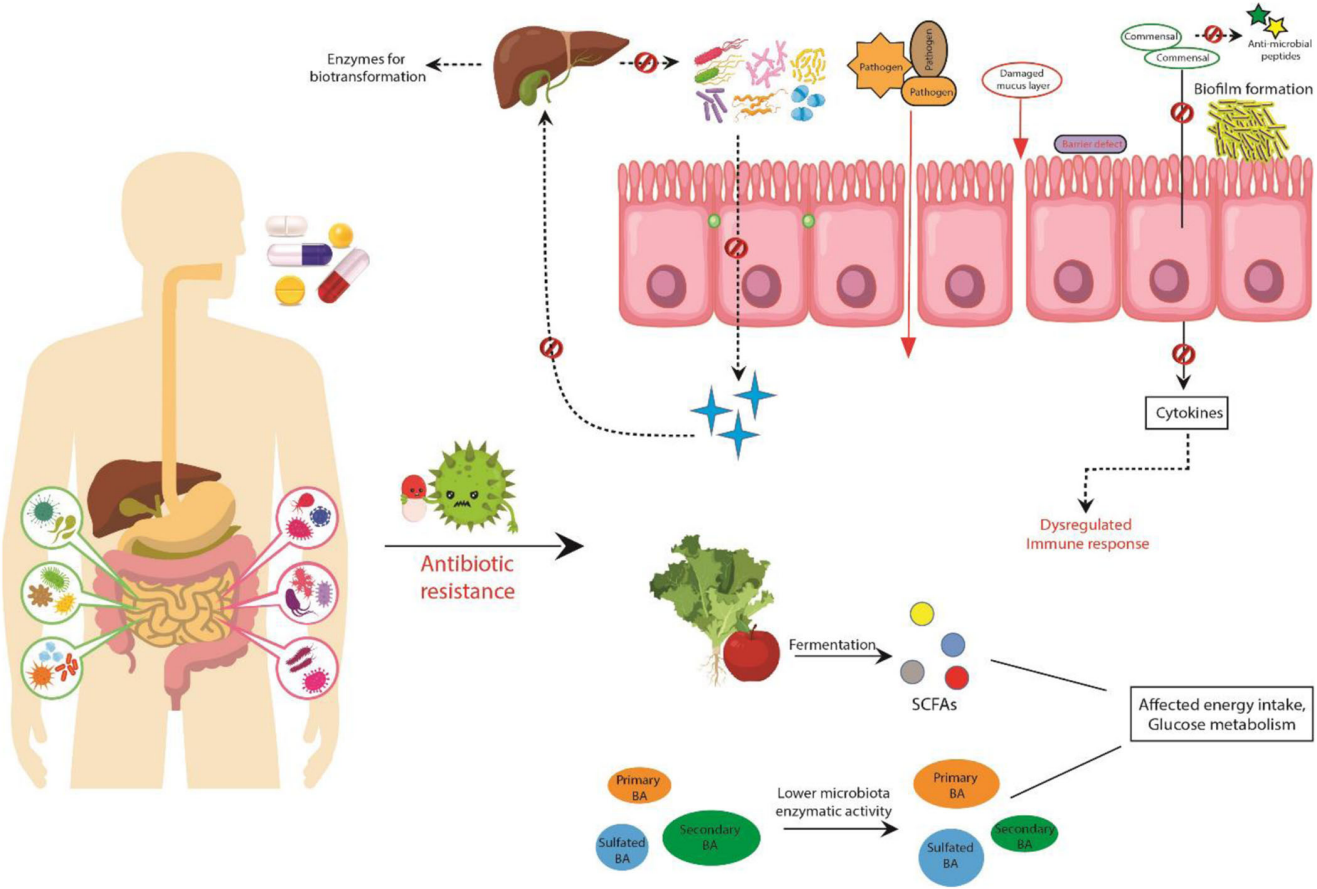
The negative impacts that can occur on host health due to overuse and misuse of antibiotics

This review thus focuses on the negative impacts of antibiotics on human health from pregnancy through to adulthood, most of which are microbiota‐dependent, although we also provide evidence of nonmicrobiota‐associated negative impacts. We discuss the changes to microbiota composition and functionality and the consequences for host health (Figure 1). We look at the impact of antibiotics at the single bacterial cell level, and how antibiotic use and misuse result in antibiotic resistance development. Further, we consider alternative approaches to antibiotic therapy and discuss therapeutics that can be used to maintain and improve host health and minimize the effects of antibiotics when used.

## 2 INTRODUCTION TO MICROBIOTA COMPOSITION FROM INFANCY TO ADULTHOOD

It was previously believed that infants are protected in the mother's womb which is a sterile environment, but studies have now demonstrated that amniotic fluid samples, placenta from mothers, and meconium samples from infants contain bacterial DNA suggesting the early exposure of infants to bacteria (Aagaard et al., 2014; de Goffau et al., 2019; Moles et al., 2013; Stinson et al., 2019). However, this is much debated (Perez‐Muñoz et al., 2017) due to issues with contamination and varying interpretations, thus we emphasize on microbiota development from infancy to adults in this review.

The gut microbiota in the early stages of life becomes more diverse until it reaches a stable adult‐like composition by 2–4 years of age. Following birth, the gut is colonized by facultative anaerobes due to the partially aerobic or microaerophilic environment. These then generate the appropriate atmosphere for the development of anaerobes by consuming the available oxygen. Thereafter, followed by early exposure to food (breast milk or infant formula), this composition changes, and facultative anaerobes such as *Bifidobacterium, Bacteroides*, and *Clostridium* dominate (Voreades et al., 2014). An initial decrease in Proteobacteria and *Enterobacteriaceae* accompanied by increases in Bacteroidetes and bifidobacteria have been reported in many studies (Bäckhed et al., 2015; Bokulich et al., 2017). The establishment of the adult‐like microbiota occurs at 2–4 years of age, which is represented by the high relative abundance of Bacteroidetes and Firmicutes (Fouhy et al., 2019).

**Figure 2:**
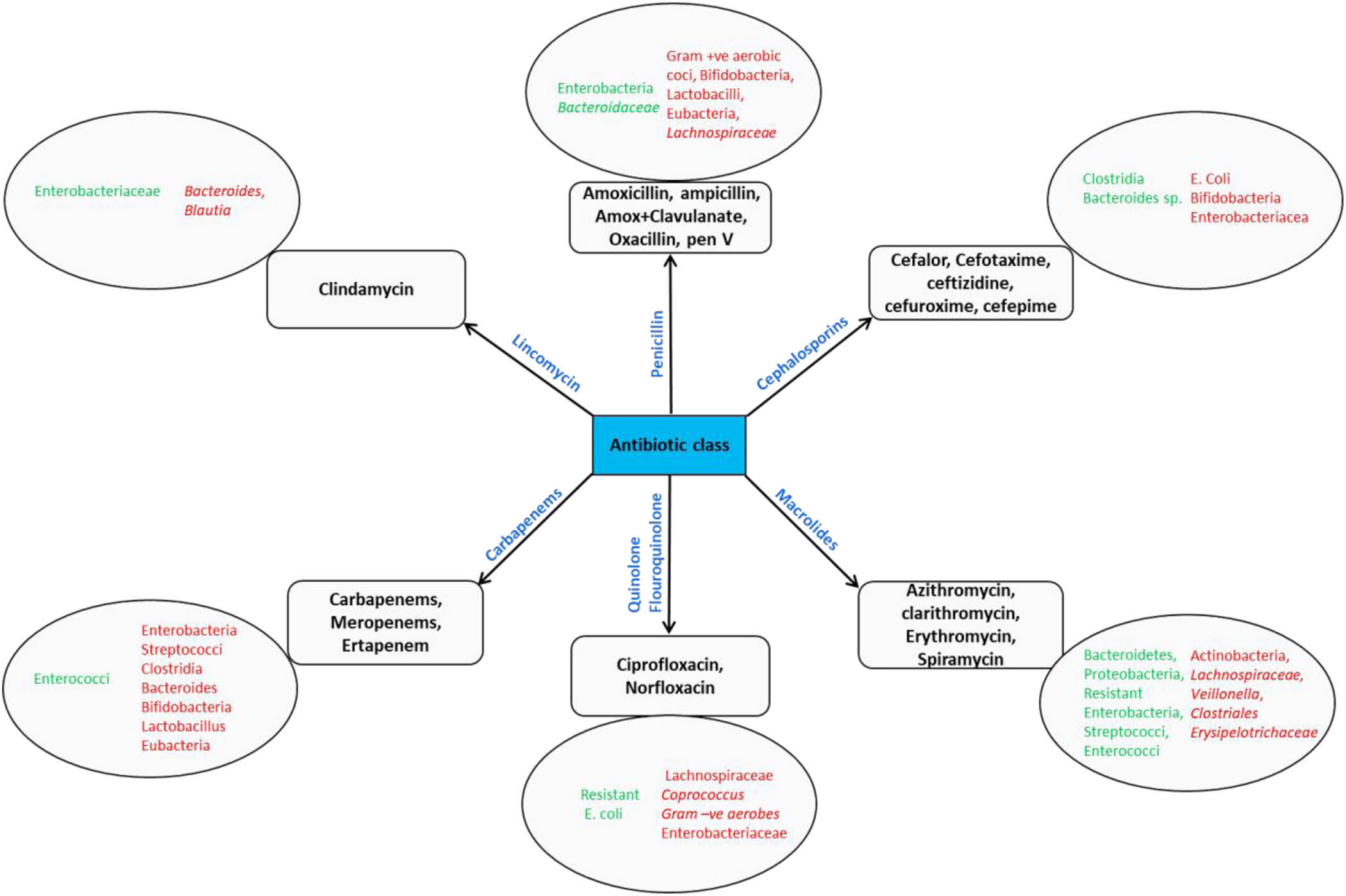
Diagrammatic representation of the effect of various antibiotics on human gut bacteria. Green (left) denotes increasing levels, while red (right) indicates decreasing levels

It is largely accepted that the mother is the most important source of the gut microbiota for infants (Asnicar et al., 2017; Ferretti et al., 2018). The establishment of the healthy infant gut microbiota and its subsequent development is a continuous process that is influenced by several factors. Mode of delivery is one of the first factors that influence the infant gut microbiota, with vaginally delivered infants having microbiota that is more diverse and similar to their mothers' vaginal microbiota while cesarean section‐born infants are deprived of this exposure and thus have gut microbiota similar to their mothers' skin and the hospital environment. These differences are significant and studies have demonstrated an increased association of *Propionibacterium, Corynebacterium, Staphylococcus, C. difficile*, and *Streptococcus* and lower abundances of bifidobacteria and *Bacteroides* with cesarean‐born neonates, whereas *Lactobacillus, Prevotella*, and *Sneathia* spp. are associated with vaginally delivered neonates (Azad et al., 2013; Dominguez‐Bello et al., 2010; Penders et al., 2006). Feeding habit is another crucial factor affecting the infant's gut microbiota composition. Because of the presence of oligosaccharides in human milk (human milk oligosaccharides) that are largely used by bifidobacteria, breastfed infants show higher levels of bifidobacteria compared to formula‐fed infants, and the proportions remain high even postweaning (Bezirtzoglou et al., 2011; Fallani et al., 2011). *Bacteroides, Streptococcus*, and *Lactobacillus* have also been reported in breastfed infants (Harmsen et al., 2000). Formula‐fed infants present higher abundances of *Escherichia coli, C. difficile*, the *Bacteroides fragilis* group, and lactobacilli than their breastfed counterparts (Penders et al., 2006). Gestational age is another factor that affects the gut microbiota composition with preterm infants showing lower diversity, higher abundance of Proteobacteria and reduced levels of obligate anaerobes such as *Bifidobacterium, Bacteroides*, and *Atopobium* compared to full‐term infants (Arboleya et al., 2012; C. J. Hill et al., 2017; Moles et al., 2013). Another factor is antibiotic administration, as shown in Figure 2, and is discussed below.

The gut microbial composition changes throughout pregnancy and a healthy pregnancy is characterized by an increase in the bacterial load and profound alterations in the composition of gut microbiota (Nuriel‐Ohayon et al., 2016). During the first to the third trimester of pregnancy, major changes such as an overall increase in Proteobacteria and Actinobacteria and reduced richness have been reported owing to the major physiological changes (Koren et al., 2012). Also, the vaginal microbiota during pregnancy has been reported to change—represented by the high bacterial load with high *Lactobacillus* abundance, low richness, and diversity compared to the vaginal microbiota of nonpregnant females (Aagaard et al., 2012; Freitas et al., 2017).

The gut microbiota of healthy adults is predominantly composed of the phyla Firmicutes and Bacteroidetes, representing the majority, followed by Actinobacteria, Proteobacteria, and Verrucomicrobia (Arumugam et al., 2011). Indeed, the gut microbiome can be perturbed by short‐term use or even low‐doses of antibiotics that can have long‐term effects on health (Jernberg et al., 2010), this cautions against the misuse and overuse of antibiotics, particularly in pregnant women and young children.

## 3 ANTIBIOTIC TYPES COMMONLY ADMINISTERED

The use of antibiotic therapy during pregnancy and lactation varies depending on the underlying condition, country, and medical guidelines, but some of the most commonly prescribed antibiotics during pregnancy are β‐lactam antibacterials (Petersen et al., 2010). Some of the other antibiotic classes prescribed include sulfonamides/trimethoprim and macrolides/lincosamides/streptogramins (de Jonge et al., 2014). Common infections for which antibiotics are prescribed during pregnancy include urinary tract infections, respiratory tract infections, skin or ear infections, bacterial vaginosis, and fever of unknown origin (Heikkila, 1993; Petersen et al., 2010). Antibiotics are frequently administered to mothers during labor to prevent Group B *Streptococcus* transmission, to reduce and prevent infections in the endometrium, and to prevent wound infections (Braye et al., 2019); though the WHO advises against the prophylactic use of antibiotics post uncomplicated delivery. This is of concern as infant antibiotic exposure through intrapartum antibiotic prophylaxis (IAP) has been shown to alter infant gut microbial diversity (Tapiainen et al., 2019). Further, antibiotics are commonly prescribed to newborns, owing to their high susceptibility to infections and lowered immunity, particularly in premature infants (Clark, 2006; Vergnano et al., 2005). The most commonly used antibiotics for infants include amoxicillin, co‐amoxiclav, benzylpenicillin, cephalosporins, gentamycin, vancomycin, clindamycin, and azithromycin. These antibiotics are indicated in respiratory and ear infections, bronchitis, pharyngitis, and high temperature (CDC, 2017). Table 1 summarizes the commonly used antibiotics and their indication for use.

**Table 1:** Summary of the various antibiotics used commonly, their mode of action, and indications when used

Antibiotic	Class of antibiotic	Broad/narrow spectrum	Mode of action	Target pathogen	Reference
Amoxicillin, ampicillin	β‐Lactam	Broad‐spectrum	Bactericidal	Rhinosinusitis, respiratory and genitourinary tract infections, septicemia	Akhavan et al. (2020); Peechakara and Gupta (2021)
Cephalosporin	β‐Lactam	Broad‐spectrum	Bactericidal	Urinary and respiratory tract infection, Gram‐negative bacteria	Bui and Preuss (2021)
Azithromycin/erythromycin	Macrolides	Broad‐spectrum	Bactericidal	Sinusitis, pneumonia, respiratory tract, and skin infection, urogenital and chlamydial infection	Pitsouni et al. (2007); Patel and Hashmi (2021)
Metronidazole	Nitroimidazole	Broad‐spectrum	Bactericidal	Protozoal infections, *C. difficile* infections, Gram‐negative bacterial infections	Rineh et al. (2014); Weir and Le (2020)
Gentamycin	Aminoglycoside	Broad‐spectrum	Bactericidal	Urinary tract infection, Gram‐negative bacterial infections	Habak and Griggs, (2020); Chaves and Tadi (2021)

## 4 IMPACT OF ANTIBIOTICS ON GUT MICROBIAL COMPOSITION

### 4.1 Impact of antibiotics during pregnancy and lactation

Perinatal and peripartum antibiotic use can impact gut microbial colonization and the resistome profile in infants (Wong et al., 2020; Zhou et al., 2020). To understand the potential impact of antibiotic administration on offspring during pregnancy, scientists examined the temporal impact of cefoperazone, on both maternal and offspring microbiota when administered during the peripartum period in an interleukin 10 (IL‐10)‐deficient murine model of colitis (Miyoshi et al., 2017). Offspring from cefoperazone‐exposed dams developed altered gut microbial communities into adulthood and had increased susceptibility to spontaneous and chemically induced colitis (Miyoshi et al., 2017). Similar results were demonstrated by Schulfer et al. (2018), who inoculated germ‐free pregnant mice with an antibiotic‐altered microbial community. They observed that the altered microbial community was transmitted to the IL‐10‐deficient offspring which resulted in the development of markedly increased colitis (Schulfer et al., 2018). Another study demonstrated that uptake of antibiotics during pregnancy can lead to alterations in the vaginal microbial composition before birth (Stokholm et al., 2014); this can impact the microbial composition infants receive at birth (Dobbler et al., 2019). Maternal antibiotic uptake during pregnancy has been reported to be associated with altered microbial composition depending on the antibiotic type (Azad et al., 2016; Coker et al., 2020). It is also associated with increased risk of asthma and allergy in the infant (Stokholm et al., 2014) although there is some controversy around this (Kim et al., 2019), as well as with functional impairment in development and cognition (Kenyon et al., 2008), obesity (Mueller et al., 2015), immunological alterations and development of diabetes (Tormo‐Badia et al., 2014) in offspring.

Many studies have demonstrated that IAP‐exposed infants in the first weeks of life have lower proportions of Actinobacteria and Bacteroidetes (Aloisio et al., 2016; Nogacka et al., 2017), high oral Proteobacteria levels (Gomez‐Arango et al., 2017), and lower levels of bifidobacteria (Mazzola et al., 2016). At 3 months, they show under‐representation of *Bacteroides, Parabacteroides*, and higher *Enterococcus* and *Clostridium* (Azad et al., 2016), as well as a higher abundance of *Enterobacteriaceae* (Mazzola et al., 2016), as compared to nonantibiotic exposed infants. Similar results were reported in a recent study including 22 newborns which demonstrated that maternal intrapartum antibiotics can affect the infant oral microbiota with phyla Actinobacteria, Bacteroidetes, and Proteobacteria being more abundant in infants of antibiotic‐treated mothers (Li et al., 2019). Another study in 28 preterm infants (with fecal samples collected on Day 7 and 14 from birth) demonstrated similar results with decreases in Bacteroidetes and *Bifidobacterium* in prenatal antibiotic‐exposed infants and suggested that the altered microbiota resembled the resistant bacteria in the neonatal intensive care unit during the same period (Zou et al., 2018). Many of these effects are similar to those observed following postnatal antibiotic administration (Tapiainen et al., 2019). Ampicillin use as an intrapartum prophylactic drug in mothers against Group B *Streptococcus* is also demonstrated to reduce the levels of bifidobacteria in infants (Aloisio et al., 2014). Similar effects of perinatal antibiotic exposure have been observed in preterm infants (*n* = 40); marked by increased levels of *Enterobacteriaceae* species and reduced levels of *Bacteroidaceae* (Arboleya et al., 2016).

Maternal antibiotic administration during lactation also influences the milk microbiota (Hermansson et al., 2019; Soto et al., 2014), which can in turn influence the infant gut microbial composition.

### 4.2 Impact of antibiotic administration directly to infants on the infant gut microbiota

Premature infants are very often treated with antibiotics owing to their health conditions, and antibiotics are one of the most commonly prescribed drugs in the NICU (Clark, 2006). Many of the prophylactic antibiotics are broad‐spectrum and thus affect a huge proportion of the gut bacterial community, leading to many alterations in the early establishment pattern. Studies have reported that very preterm infants who received prolonged antibiotic treatment had less diverse bacterial populations and reduced species richness in their gut and more antibiotic resistance genes (ARGs; Gasparrini et al., 2019; Gibson et al., 2016). Both short‐term and long‐term exposure of preterm infants to antibiotics can alter their gastrointestinal microbiota. These changes include decreases in bifidobacteria and *Bacteroidetes* relative abundance, and an increase in *Enterococcus* abundance (Gasparrini et al., 2019; Zou et al., 2018; Zwittink et al., 2018). Extended antibiotic treatment in premature infants can result in an increased risk of developing late‐onset sepsis (primarily caused by Group B *Streptococcus*), necrotizing enterocolitis (NEC), and overall mortality (Esaiassen et al., 2017; Kuppala et al., 2011).

Amoxicillin treatment in infants (*n* = 31) for 7 days was shown to completely eradicate *Bifidobacterium adolescentis* species which was accompanied by decreased diversity of the bifidobacteria population (Mangin et al., 2010). In a cohort of 18 infants, we have previously found that the use of ampicillin and gentamicin in early life resulted in higher levels of Proteobacteria and lower proportions of *Bifidobacterium* and *Lactobacillus* 4 and 8 weeks after the treatment (Fouhy et al., 2012). A study reporting on the effects of administering the broad‐spectrum antibiotic, cefalexin, in 26 infants, in the first 4 days of life revealed that the gut microbiota of antibiotic‐treated infants showed less diversity than the control antibiotic‐free infants (Tanaka et al., 2009). The antibiotic also arrested the growth of some bacterial groups such as bifidobacteria and resulted in unusual colonization of *Enterococcus* in the first week. Further to this, a high *Enterobacteriaceae* population was observed 1 month following antibiotic treatment as compared to the control antibiotic‐free infants (Tanaka et al., 2009). Antibiotics change the dominant members of the bacterial community which could have profound effects on immune development, metabolism, and growth of the infant (Cox et al., 2014; Nobel et al., 2015).

Similar results of reduced gut microbiota diversity have been observed in children (*n* = 39, monthly sampling in the first 3 years of life) who have undergone antibiotic therapy. The authors also reported the presence of ARGs on mobile genetic elements long after cessation of treatment (Yassour et al., 2016). Antibiotic use during infancy and childhood has been associated with the altered microbial composition and metabolic functions (Korpela et al.,^2016^), higher risk of asthma and allergy development (Ni et al., 2019; Yamamoto‐Hanada et al., 2017), and obesity (L. C. Bailey et al., 2014) in later life.

### 4.3 Impact of antibiotics on the gut and oral microbiota in adults

To investigate the long term effects of antibiotics on the healthy state microbial composition, the antibiotics amoxicillin (*n* = 6; Cochetière et al., 2005), ciprofloxacin (*n* = 3; Les Dethlefsen et al., 2008), and cefprozil (*n* = 24; Raymond et al., 2016) were administered to healthy individuals. These studies reported changes in microbial composition persisting for up to 12 weeks after treatment had ended with the incomplete restoration of microbial composition and emergence of antibiotic‐resistant strains. In one study, the authors examined the distal gut microbiota of three individuals over 10 months post‐administration of the antibiotic ciprofloxacin. They reported that the effect of ciprofloxacin on the gut microbiota was profound and rapid with a decrease in richness and diversity of microbiota accompanied by shifts in levels of Bacteroidetes, *Lachnospiraceae*, and the *Ruminococcaceae*. By 1 week after the end of each course, communities began to return to their initial state, but the return was incomplete and variable from the initial stage (Dethlefsen & Relman, 2011). Many studies have investigated the long‐term impact on gut microbiota following a course of antibiotics. A short term course of clindamycin (7 days) resulted in significant disturbances in the bacterial community such as a sharp decline in *Bacteroides* (Jernberg et al., 2007; Löfmark et al., 2006) and enterococcal colonies (Lindgren et al., 2009) that remained for up to 2 years post‐treatment and was accompanied by increased levels of ARGs and strains (Jernberg et al., 2007; Lindgren et al., 2009; Löfmark et al., 2006). Another study (*n* = 10) that analyzed adult fecal samples from 11 days to 12 months post antibiotic treatment demonstrated that the use of ciprofloxacin for 10 days resulted in lower abundances of *Bifidobacterium* but did not affect *Lactobacillus* and *Bacteroides* levels, but clindamycin treatment for the same number of days caused *Lactobacillus* and *Bifidobacterium* to decrease and *Bifidobacterium* did not normalize until 1‐year post‐treatment, showing that different bacterial groups require different times for normalization post‐treatment (Rashid et al., 2015).

Pérez‐Cobas and Gosalbes et al. (2013) also reported that the type of antibiotic, whether static or cidal, had varying effects on the gut microbiota which was reflected at the functional level. For instance, in a study of four subjects, a bacteriostatic drug resulted in the flourishing of Gram‐negative bacteria which was related to an increase in the number of genes involved in lipopolysaccharide (LPS) synthesis, while the bactericidal drug was associated with an increase in Gram‐positive bacteria which was accompanied by an over‐representation of genes involved in endospore formation (Pérez‐Cobas & Artacho et al., 2013). Antibiotic administration for the eradication of *Helicobacter pylori* demonstrated that the antibiotics could affect the indigenous microbiota and lead to the development of resistant strains which can persist for years after treatment (Jakobsson et al., 2010; Sjölund et al., 2003).

Furthermore many antibiotics are used for dentistry procedures routinely. These antibiotics can increase the number of resistant strains present orally, can increase the minimum inhibitory concentrations, and can also eliminate the nonpathogenic strains (Harrison et al., 1985; Ready et al., 2004), which can lead to systemic infections and inflammation.

## 5 CONSEQUENCES OF ANTIBIOTIC‐INDUCED MICROBIOTA CHANGES FOR HEALTH AND DISEASE

### 5.1 In adults

Due to the role of the microbiota in host metabolism and physiology, many studies postulate that microbial imbalances can be related to obesity (Riley et al., 2013; Scott et al., 2016), diabetes (Boursi et al., 2015; Mikkelsen et al., 2015), and asthma (Arrieta et al., 2015; Kozyrskyj et al., 2007). Blaser and Falkow (2009) have suggested a link between the “missing microbes” and modern conditions such as obesity and juvenile diabetes. These multifactorial conditions can be controlled by identifying the controllable factors such as the microbiota component and dietary habits, thus preventing them from occurring if possible.

Studies have reported a link between antibiotic usage and obesity (Del Fiol et al., 2018). Some studies suggest that an increased ratio of Firmicutes to Bacteroidetes rather than specific levels is associated with obesity (Kasai et al., 2015), though results are conflicting (Duncan et al., 2008; Schwiertz et al., 2010). While the microbial component of obesity is debated, studies have reported a common change at functional microbial levels. Indeed, obese individuals have higher short‐chain fatty acid (SCFA) content compared to lean individuals (Schwiertz et al., 2010; Turnbaugh et al., 2006). Furthermore, obesity is associated with metabolic alterations related to glucose homeostasis and insulin resistance and linked to the development of diabetes (Cani et al., 2012). In a study in 96 humans (48 each antibiotic group and controls), researchers reported significant and persistent weight gain after an episode of infectious endocarditis in patients who had been treated with vancomycin and gentamycin (Thuny et al., 2010).

An association between antibiotic‐induced changes in microbial colonization and type 1 diabetes in male mice was reported (Candon et al., 2015). A combination of broad‐spectrum antibiotics or vancomycin alone was given to neonatal nonobese diabetic mice that spontaneously developed autoimmune type 1 diabetes. The microbiota was significantly altered with an increase in *Escherichia* and *Lactobacillus* species and a decrease of the Clostridiales order compared to controls. A major reduction of IL‐17‐producing cells was also observed in the lamina propria of the ileum and the colon of vancomycin‐treated mice (Candon et al., 2015), which can affect host defense mechanisms. Some studies in human populations also suggest a link between repeated use of broad‐spectrum antibiotics and diabetes (Boursi et al., 2015; Mikkelsen et al., 2015), while some suggest a protective and preventative role of antibiotics and diet in diabetes development in diabetes‐prone animals partly due to lowering of specific antigenic load or development of tolerogenic APCs (Brugman et al., 2006; Hu et al., 2015).

Associations between altered microbial composition and type 2 diabetes are more established, with decreased levels of butyrate‐producing bacteria reported in type 2 diabetic patients (Gurung et al., 2020). X. Zhang et al. (2013) studied 121 subjects with normal glucose tolerance, prediabetes, and newly diagnosed diabetes and reported that there is modulation of the gut microbial composition at the prediabetes stage which can act as a marker for the development of diabetes state.

Antibiotics can lead to antibiotic‐associated diarrhea (AAD) and studies have demonstrated that clindamycin can result in alteration of the microbial community which can promote the colonization of potential pathogens such as *C. difficile* which can lead to diarrhea and colitis (Buffie et al., 2012; McDonald, 2017). Another study in a mouse model reported that antibiotic treatment resulted in decreased alpha and beta diversity, which potentially caused a decrease in levels of serotonin, tryptophan hydrolase, and secondary bile acids which can further affect gut motility and metabolism (Ge et al., 2017).

### 5.2 During pregnancy and infancy

Extrinsic factors such as antibiotics can alter the diversity of the maternal microbiota that can affect the infant's gut microbiota diversity, immunity, and disease development in later life, both directly and indirectly (Azad et al., 2016; Nyangahu et al., 2018; Tapiainen et al., 2019; Tormo‐Badia et al., 2014). According to the hygiene hypothesis, if the host is not exposed to a diverse range of microbiota early in childhood or in the developing stages, immune‐related disorders may develop such as asthma and allergic sensitization. Antibiotics can have a similar effect when administered during infancy. Preterm infants are often exposed to antibiotics which results in an altered microbial composition (Clark, 2006; Gibson et al., 2016; Zou et al., 2018), predisposing them to probable infections such as NEC, and invasive fungal infections (Esaiassen et al., 2017). Animal studies have reported that administration of low dose or subtherapeutic concentrations of antibiotics in early life can disturb the microbial composition, affecting the expression of genes involved in immunity and carbohydrate metabolism, and can alter metabolic homeostasis predisposing the host to adiposity later in life (Cho et al., 2012; Cox et al., 2014; Schulfer et al., 2019). Obesity has been widely linked with altered microbial colonization during early life owing to the role of the gut microbiota in dietary metabolism. Studies have demonstrated that early‐life antibiotic exposure has potential links to increases in body mass index, overweight and central adiposity, and this can be gender‐specific affecting males more than females (Azad et al., 2014; Murphy et al., 2014). Both murine models (Cho et al., 2012; Cox et al., 2014) and human studies have reported that antibiotic exposure in the first few months of life is associated with increases in body mass index and risk of overweight (L. C. Bailey et al., 2014; Scott et al., 2016; Trasande et al., 2013) and asthma (Kozyrskyj et al., 2007; Risnes et al., 2011) in childhood. Similarly, studies have reported that antibiotic administration in early life can be associated with a heightened risk of asthma, allergy, and atopic dermatitis, and IBD (Johnson et al., 2005; Kronman et al., 2012; Ni et al., 2019; Yamamoto‐Hanada et al., 2017). NEC and AAD have also been associated with prolonged or prophylactic antibiotic uptake in early life (Alexander et al., 2011; Kuppala et al., 2011; Michael Cotten et al., 2009).

### 5.3 Changes in immune response

The immune system is trained to fight pathogens during infancy, and this is the time when microbial colonization takes place. Any disturbance to microbial colonization has been shown to affect immune maturation due to this co‐developmental process. Studies in germ‐free mice have confirmed that the absence of microbes in the gut results in both physiological and immunological changes to the gut environment. These changes include alterations in mucus thickness and composition (Szentkuti et al., 1990), reduced gastric motility (Abrams & Bishop, 1967), improper development and functioning of intestinal cells and immune cells (Cahenzli et al., 2013; Vaishnava et al., 2008) and improper development of the immune system (Bauer et al., 1963; Macpherson & Uhr, 2004). Antibiotic treatment has been shown to reduce the thickness of the colonic mucus layer thus increasing the risk of pathogen invasion and intestinal inflammation in 8–10‐week‐old mice (Wlodarska et al., 2011). Another study in mice reported that antibiotic‐induced alterations in the microbiota shift the TH1/TH2 balance toward TH2‐dominant immunity—which leads to atopy development, accompanied by a reduced number of lymphocytes (Oyama et al., 2001). Altered microbial composition and altered gene maturation profile such as downregulation of genes coding for MHC class 1b and class II proteins and products of Paneth cells such as defensins were seen post clamoxyl treatment in neonatal rats, which could affect mucosal barrier development (Schumann et al., 2005).

A study demonstrated that certain molecules produced by bacteria in the gut are involved in immune system maturation. Indeed, germ‐free mice colonized with *B. fragilis* producing a bacteria polysaccharide resulted in correcting T‐cell‐deficiencies and improving T(H)1/T(H)2 imbalances along with promoting lymphoid organogenesis; this was not observed in the case of the nonpolysaccharide‐producing mutant *B. fragilis* (Mazmanian et al., 2005). Studies have reported that the secretion of antimicrobial peptides by intestinal epithelial cells is regulated by the microbiota in the microenvironment. Germ‐free mice colonized with conventional or human microbiota or specific probiotic species or LPS demonstrated increased production of antimicrobial peptides like REGIII‐γ, boosting the innate immune response (Brandl et al., 2008; Cash et al., 2006; Natividad et al., 2013).

A study in mice demonstrated that prenatal antibiotics not only altered the pattern of microbiota colonization in infant mice but also negatively affected the activity of CD8+ T lymphocytes towards viral infections affecting their immune responses (Gonzalez‐Perez et al., 2016). Further, they also observed that infant mice were more susceptible to infection when born in stricter hygienic facilities (Gonzalez‐Perez et al., 2016). Similar results were reported post‐antibiotic treatment in another murine model with low bacterial diversity alongside reduced cytokine production by CD4+ T lymphocytes and reduced production of interferon‐γ (D. A. Hill et al., 2010). In a study involving influenza patients, the authors reported that subjects with low titers of infection and treated with antibiotics had low immune responses accompanied with microbiota loss, low immunoglobulin (Ig) G1, IgA, and secondary bile acid levels against the infection (Hagan et al., 2019).

These studies demonstrate the complex relationship between the microbiota and the host immune response, and the impact of antibiotics on this interaction which needs to be further studied. It can also impact the effectiveness of vaccines used postantibiotic treatment.

## 6 INFLUENCE OF ANTIBIOTIC‐INDUCED CHANGES ON MICROBIOTA FUNCTIONALITY AND BACTERIAL BEHAVIOR AT THE SINGLE‐CELL LEVEL

### 6.1 Changes in metabolites

The gut microbiota is responsible for the production of many essential metabolites including SCFAs and amino acids (Mills et al., 2019). Studies have reported that commensal‐produced butyrate and propionate have anti‐inflammatory roles, promoting the generation and differentiation of regulatory T cells (Arpaia et al., 2013; Furusawa et al., 2013), with roles in energy metabolism (den Besten et al., 2013; De Vadder et al., 2014). By impacting the composition of the microbial community, antibiotics also alter microbiota functionality and thus the metabolites produced (Ferrer et al., 2017).

For example, metabolomics profiles were analyzed in antibiotic‐treated piglets fed a corn‐soy basal diet with or without in‐feed antibiotics from postnatal days 7–42. The antibiotic‐treated group had higher concentrations of metabolites associated with amino acid metabolism, decreasing the concentration of amino acids. A reduction in SCFA production was also reported as levels of butyrate and propionate were decreased (Mu et al., 2017). Another study in piglets (Days 7–21) demonstrated that fecal microbiota transplant (FMT) and antibiotic (amoxicillin) treatment both resulted in lowering of fatty acid oxidative catabolism and amino acid biosynthesis, though antibiotics had a more significant effect (Wan et al., 2019).

Multiple studies performed in mouse models have elucidated the effects of antibiotic treatment on host metabolic functions. A study in antibiotic (streptomycin)‐treated mice reported that antibiotic administration can affect pathways of hormone synthesis such as steroids and eicosanoids, along with altering the pathways involved in sugar, bile acids, and amino acid metabolism, thus suggesting the role of the microbiota in these pathways (Antunes et al., 2011). Sun et al. (2019) reported that mice treated with antibiotics (enrofloxacin, vancomycin, and polymixin B sulfate) showed upregulated gene expression of various cytokines in the colon, with significant metabolic shifts in valine, leucine, and isoleucine biosynthesis pathways. These alterations correlated to changes in microbial composition. One study reported that clindamycin treatment in mice resulted in significant changes in metabolite composition (30% of the compounds analyzed); the restoration of which was associated with recovery of the altered microbiota (Jump et al., 2014). Zhao et al. (2013) reported that antibiotic treatment altered the products of bacterial metabolism. This included decreased levels of SCFAs, amino acids (correlated with the abundance of *Prevotella, Alistipes*, and *Barnesiella*), and increased precursors like bile acids and oligosaccharides (associated with high levels of facultative anaerobic bacteria, *Enterococcus faecalis, Enterococcus faecium*, and *Mycoplasma*; Zhao et al., 2013). Microbial depletion in mice due to antibiotic uptake decreased baseline serum glucose levels, improved insulin sensitivity (Zarrinpar et al., 2018), altered systemic glucose metabolism along with changes in expressions of the genes in the liver and ileum involved in glucose and bile acid metabolism (Rodrigues et al., 2017). This was reported to be accompanied by reduced levels of SCFAs and secondary bile acid pools (Zarrinpar et al., 2018). Similar results were reported following vancomycin treatment (Vrieze et al., 2014). This can lead to impairment of barrier function (Kelly et al., 2015); act as a causative factor in the development of ulcerative colitis (Machiels et al., 2013), and *Salmonella* infection (Gillis et al., 2018).

Studies have also reported that antibiotic uptake can result in changes in protein expression, energy metabolism in the microbiota, with a slight increase following antibiotic therapy, which may be as a coping mechanism to antibiotic stress but decreased at later stages and post‐antibiotic usage (Pérez‐Cobas & Artacho et al., 2013). Another study demonstrated that antibiotics have a sex‐dependent effect on host metabolism. They reported that vancomycin and ciprofloxacin–metronidazole treatment resulted in significant reductions of Firmicutes and SCFAs in female mice, which was only observed after vancomycin treatment in males. They also reported that both antibiotic exposures significantly decreased the levels of alanine, branched‐chain amino acids (leucine, isoleucine, and valine), and aromatic amino acids in colonic contents of female mice but not in male mice (Gao et al., 2019).

### 6.2 Accumulation of metabolites/xenobiotics

**Figure 3:**
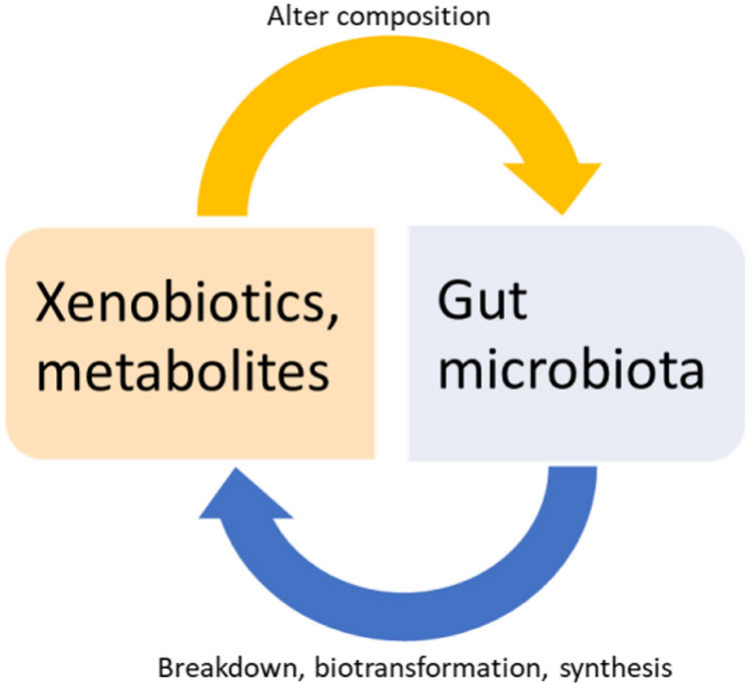
Figure demonstrating cyclical relation between gut microbiota, xenobiotics, and metabolites

Xenobiotics (including antibiotics, heavy metals, and environmental chemicals) have an impact on gut microbial composition. The effect here is cyclical in that the microbiota is necessary for xenobiotic biotransformation (Figure 3). The metabolism of xenobiotics before it reaches its target organ site is largely dependent on the microbiota. The gut microbiota can affect the xenobiotic half‐life in the host, the extent to which they reach the target receptor, and may also influence the host's capacity to metabolize xenobiotics (Koppel et al., 2017). Both in vivo and in vitro studies have shown that the gut microbiota is involved in the biotransformation of xenobiotics (Lu et al., 2013). Studies using germ‐free and conventional rodents reported that the absence of gut microbiota‐affected gene expression of many liver enzymes including active androstane receptor and host detoxifying enzymes such as glutathione peroxidases, sulfotransferase, epoxide hydrolases, and *N*‐acetyltransferases (Björkholm et al., 2009; Meinl et al., 2009). However, the absence of microbes necessary for metabolizing particular compounds can result in their accumulation in the host which may lead to toxicity. Ciprofloxacin administration to SPF and germ‐free mice revealed decreases in hepatic Cyp3a11 expression, this was associated with the gut microbiota. The lower lithocholic acid (LCA) levels due to decreases in LCA‐producing bacteria post antibiotic administration could be responsible for decreases in the expression of Cyp3a11. Similar effects in humans can result in less clearance of multiple CYP3A4 (human analog of Cyp3a11)‐dependent medications (Lynch & Price, 2007).

Another study speculated that increased levels of hepatic lipid accumulation and TG levels in mice were due to antibiotic treatment and the altered gut microbial composition (Jin et al., 2016). Studies have shown that antibiotic treatment in mice increases the levels of sialic acid and succinate which increase the susceptibility of the host to *Salmonella* and *C. difficile* infections (Ferreyra et al., 2014; K. M. Ng et al., 2013). In the same manner, an antibiotic‐altered microbial composition can result in deficiencies of certain metabolites or vitamins that are solely produced by bacteria. For example, one study reported that antibiotic‐induced changes in the gut microbiota of mice resulted in shifts in copper (Cu) metabolism. This can have consequences for immunity and the intestinal barrier due to the role of Cu in these functions (Miller et al., 2019).

Furthermore, cross‐feeding is a significant feature of the gut microbiota. For example, *B. adolescentis* produces lactate and acetate by utilizing fructo‐oligosaccharides and starch. Butyrate‐producing anaerobes cannot utilize fructo‐oligosaccharides and starch but rely on lactate and acetate as growth substrates. Therefore, *B. adolescentis* indirectly facilitate the proliferation and expansion of butyrate‐producing species through cross‐feeding. This type of dependency is also seen in many other bacterial groups (Heinken & Thiele, 2015; Rowland et al., 2018). This codependency can be disturbed by antibiotic use leading to increased accumulation or deficiency of some metabolites/compounds. For example, a study reported a decrease in abundance of Gram‐negative bacteria in the gut by vancomycin which is a Gram positive‐targeting antibiotic (Ubeda et al., 2010). This can be due to the interdependency of bacteria in their community.

### 6.3 Changes in bacterial signaling pattern

Antibiotics can alter the transcription of several major functional genes such as those encoding transport proteins, genes involved in the metabolism of carbohydrates, and protein synthesis (Goh et al., 2002; J. T. Lin et al., 2005). A study demonstrated induced expression of virulence‐associated genes in *Pseudomonas aeruginosa* leading to higher secretion of rhamnolipids and phenazines on exposure to subinhibitory concentrations of antibiotics (Shen et al., 2008). Many studies have reported that aminoglycosides (Hoffman et al., 2005), β‐lactams (Kaplan et al., 2012; K. M. Ng et al., 2014), vancomycin, and oxacillin (Mirani & Jamil, 2010) can induce biofilm formation even at sublethal concentrations. These biofilms then act as reservoirs of antibiotic resistance. This confers additional resistance to bacteria against several antibiotics and host defense, which can make treatment difficult in humans and can cause several issues such as blockage of pipes/equipment in healthcare settings and food industries (Muhammad et al., 2020).

Bacteria interact with their host using pattern recognition receptors (PRR) via signaling through the production of bile acids, SCFAs, fatty acids, amino acids, LPS, lipoteichoic acid, flagellin, CpG DNA, and peptidoglycan. These signaling molecules either serve as a source of energy for other cells, regulate or modulate the function of immune cells such as monocytes, macrophages, T cells via G‐protein coupled receptor and nuclear receptor family, free fatty acid receptors (Brestoff & Artis, 2013). Antibiotic use results in a reduction of these bacteria and hence the PRRs such as TLR signaling and downstream regulation of innate defenses (Willing et al., 2011). A study reported that an antibiotic‐mediated decrease in butyrate‐producing bacteria resulted in reduced epithelial signaling through the intracellular butyrate sensor peroxisome proliferator‐activated receptor γ (Byndloss et al., 2017). Similarly, antibiotic disruption of the commensal microbiota in newborn mice increases their susceptibility to pneumonia, due to interrupted migration of IL‐22 producing lymphoid cells (IL‐22 + ILC3; Gray et al., 2017). This effect could be reversed by the transfer of commensal microbiota to mice at birth (Gray et al., 2017). Another study reported the importance of commensals in protecting against colon injury and maintaining intestinal homeostasis (Rakoff‐Nahoum et al., 2004). In this case, commensals induced release of protective factors via TLRs; these factors were not released in mice treated with antibiotics lacking the commensal bacteria (Rakoff‐Nahoum et al., 2004). Antibiotics can thus impact complex host‐microbial interactions due to changes in microbial community composition.

### 6.4 ARG reservoir

ARGs are now reported to be found in the environment including oceans and freshwater bodies (Hatosy & Martiny, 2015), soil (Cycoń et al., 2019), glaciers (Segawa et al., 2013), the food chain, and also within humans. Apart from bacteria, viruses have also been reported to be carriers of ARGs (Debroas & Siguret, 2019). Some of this spread of ARGs is historical such as in some untouched/uncontaminated environments (Van Goethem, 2018) but much of it is because of the wide use of antibiotics by humans.

For instance, apart from their use in treating infections in humans, antibiotics have been widely used as growth promoters for weight gain in animals (Butaye et al., 2003) and to treat and control infections (Ding et al., 2014; Mcmanus et al., 2002). They have also been used in aquaculture for similar reasons (Cabello, 2006; Lulijwa et al., 2019). Some of these antibiotics are the same as, or are structurally similar to the ones used to treat human infections such as erythromycin, gentamycin, enrofloxacin, neomycin, streptomycin (Marshall & Levy, 2011). The development of antibiotic‐resistant bacteria in agriculture and aquaculture is a serious concern as such bacteria can enter humans through the food chain promoting cross‐resistance and also reducing the susceptibility of infectious bacteria to antibiotic treatment.

Humans are reservoirs of ARGs (J. K. Bailey et al., 2010; Clemente et al., 2015; Sommer et al., 2010) and the gut has been pinpointed as the epicenter of antibiotic resistance (Carlet, 2012). Intriguingly, some studies have reported the presence of ARGs in humans from remote communities who have had very limited exposure to antibiotic therapy. They reported high levels of acquired resistance to antibiotics such as tetracycline, ampicillin, trimethoprim/sulfamethoxazole, streptomycin, and chloramphenicol (Bartoloni et al., 2009). Similarly, ARGs in healthy humans have been reported despite the absence of antibiotic use (Bengtsson‐Palme et al., 2015; Sommer et al., 2009; de Vries et al., 2011). Studies in healthy infants and children who have never been exposed to antibiotics report the presence of genes that confer resistance to β‐lactams, fluoroquinolones, tetracycline, macrolide, sulfonamide, or multiple drug classes. The major ARG carriers were found to be *Enterococcus* spp., *Staphylococcus* spp., *Klebsiella* spp., *Streptococcus* spp., and *Escherichia*/*Shigella* spp. (Casaburi et al., 2019; Karami et al., 2006; Moore et al., 2013; L. Zhang et al., 2011).

In the gut, bacteria can horizontally and vertically transmit genes to other related or unrelated bacteria due to their proximity via mobile genetic elements (Table 2). ARGs in the infant gut microbiota can be derived from that of their mothers, as AR bacteria can be transmitted from mother to infant through breastfeeding (Parnanen et al., 2018). The presence of ARGs in humans is a global crisis because it makes the treatment of infections more difficult, costly, and inefficient.

**Table 2:** Various mobile genetic elements that can be used for the transfer of ARGs

Mobile elements and transfer of resistance genes		
Vehicle element	Description	Some antibiotic resistance genes transported
Plasmids; Rozwandowicz et al. (2018)	Extrachromosomal material	Colistin resistance, extended‐spectrum β‐lactams, β‐lactams, aminoglycoside, quinolone, sulfonamides, tetracycline, chloramphenicol, trimethoprim
Integrons; Partridge et al. (2009)	Genetic elements with site‐specific recombination system	Sulfonamide resistance (sulI), aminoglycosides, β‐lactams, quinolones, chloramphenicol, and trimethoprim
Transposons; Lupski (1987)	Mobile elements that need integration into chromosome or plasmid	β‐Lactams, macrolides, aminoglycoside, chloramphenicol, tetracycline

## 7 NONMICROBIOTA‐ASSOCIATED EFFECTS OF ANTIBIOTICS

### 7.1 In pregnancy

Associations between antibiotic usage during pregnancy with neonatal and congenital abnormalities have been reported. For example, studies have reported an increased risk of developing cerebral palsy, epilepsy, cardiac and genital malformations in infants born to mothers treated with macrolides during pregnancy and which are more harmful when consumed during the first trimester (Källén & Danielsson, 2014; Kenyon et al., 2008; Meeraus et al., 2015). Similarly, amoxicillin use during the first trimester was linked to cleft lip and cleft palate development, and though reported in only a small number of cases, it exemplifies the adverse effects of antibiotic use during pregnancy (Lin et al., 2012). Birth defects such as microphthalmia, hypoplastic left heart syndrome, atrial septal defects, and cleft lip with cleft palate have also been associated with the use of sulfonamides and nitrofurantoin during the first trimester of pregnancy (Crider et al., 2009). Similarly, trimethoprim–sulfonamide use during pregnancy is associated with a higher risk of cardiovascular malformations (Czeizel et al., 2001). The evidence in all cases is mixed (Muanda & Sheehy, 2017), and is usually linked with consumption during the early months of pregnancy. This could be due to the antibacterial impacting the neonate during the organogenesis and early development stages in utero. Though the examples cited represent potential associations and not causal relationships, a reappraisal of antibiotic usage during pregnancy is warranted.

### 7.2 General effects

Studies have reported that antibiotics can exert direct toxic effects on host tissues such as mitochondrial damage, suppressed ribosomal gene expression (Morgun et al., 2015), and oxidative tissue damage in mammalian cells (Kalghatgi et al., 2013). Though controversial, some studies suggest that antibiotic use can be related to increased risk of breast cancer (Tamim et al., 2007; Velicer et al., 2004) and increased risk of miscarriage (Fan et al., 2019). Sometimes, antibiotics can worsen the condition they are meant to treat. The bactericidal action of many β‐lactam antibiotics has been reported to increase toxin production such as Shiga toxin which is released from entero‐hemorrhagic *E. coli*, predisposing the host to a higher risk of a hemolytic uremic syndrome (Kimmitt et al., 1999; Wong, 2000). Antibiotics can also affect host metabolism directly without microbes as a mediator while making the targeted pathogen less susceptible to the antibiotic. These changes in host metabolites are mostly local to the site of infection and include high levels of AMP which decrease the efficacy of antibiotics and also increase phagocytic activity. This was accompanied by impaired immune function because of the inhibitory effect of antibiotics on the respiratory activity of immune cells (Yang et al., 2017).

## 8 ANTIBIOTIC ALTERNATIVES AND USE OF PROBIOTICS FOR RESTORING THE MICROBIAL COMMUNITY AND BETTERMENT OF HEALTH

**Figure 4:**
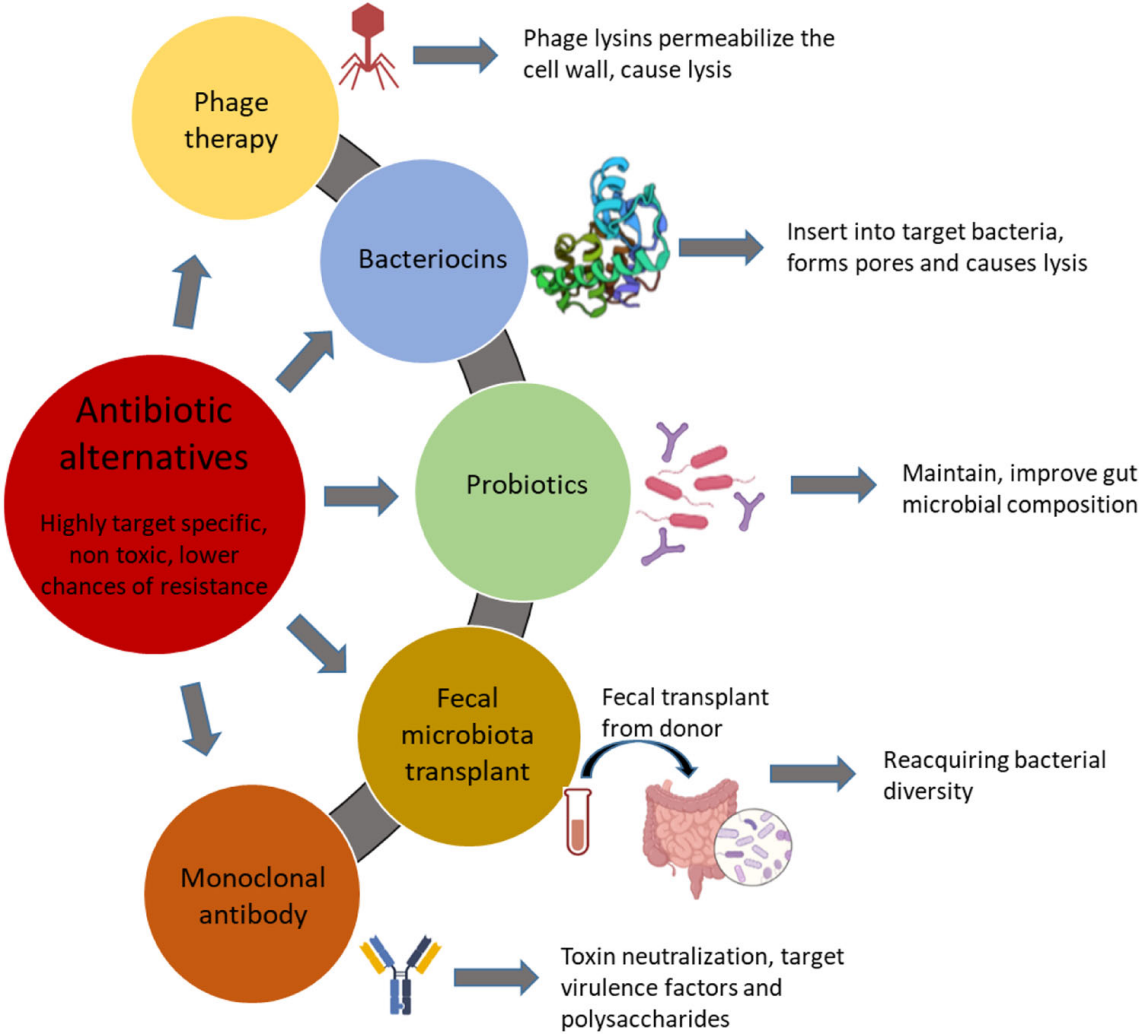
Various alternatives to antibiotics that can be used alone or in some cases in combination with antibiotic treatment

It is now well established that antibiotic use results in changes in microbial composition, the consequences of which can be detrimental for the host. Certain approaches can be used along with or post antibiotic therapy to restore the microbial composition faster (Figure 4). Probiotics are widely used for this purpose and have been shown to increase the abundance of beneficial microbes, stabilize the microbial community and thus alleviate the effects of antibiotics (Ki Cha et al., 2012; Korpela et al., 2018). Probiotics exert their effects by promoting antimicrobial peptide production, producing bacteriocins, suppressing the growth of non‐commensals via competing for nutrients and receptors on the intestinal mucosa, enhancing barrier function in the gut, and modulating immunity (Bron et al., 2011; Cazorla et al., 2018; Collado et al., 2007; O'Shea et al., 2012; Xue et al., 2017), but the use of probiotics may not lead to complete restoration of the gut microbiota.

FMT can be more beneficial for regaining microbial balance in the gut (Suez et al., 2018). FMT has been widely used therapeutically for rebalancing the microbiota of *C. difficile*‐infected patients; restoring their microbial and metabolic activity (Weingarden et al., 2014). FMT can also utilize donor fecal material from the patient itself before antibiotic therapy, known as autologous FMT. Many factors such as efficacy, cost, and suitability make FMT an attractive option but detailed studies are needed to optimize the process and understand other probable therapeutic applications beyond gut disorders (Allegretti et al., 2019; Ramai et al., 2019).

One of the major issues of antibiotic use is the development of bacterial resistance. Alternatives such as bacteriophage (phage) therapy and bacteriocins are now being investigated as substitutes for antibiotics or complementary therapies with antibiotics to overcome the issue of resistance. Phage therapy was first described and used in the early 1900s, but due to the introduction of antibiotics, phage therapy was dismissed in western medicine (D. M. Lin et al., 2017), although it is still practiced in certain regions of the world including Georgia, Russia, and Poland (C. Hill et al., 2018). With the growing antibiotic resistance crisis, phage therapy is now being revisited. The use of phage therapy to reduce biofilm and treat lung infections has been studied in mouse models and it has been demonstrated that phage therapy successfully treats respiratory *P. aeruginosa* infection (F. Cao et al., 2015; Fong et al., 2017; Waters et al., 2017). A report based on using personalized phage therapy for treating a patient with multidrug resistant *A. baumannii* reported clearance of infection and success of the treatment (Schooley et al., 2017). Furthermore, case reports of patients with bacterial prostatitis and septicemia and acute kidney injury reported pathogen eradication and decrease in clinical symptoms post phage therapy (Jennes et al., 2017; Letkiewicz et al., 2009). Another study reported that 67 of 96 patients' wounds and ulcers healed post phage therapy; healing was associated with a reduction in pathogens (Markoishvili et al., 2002). The use of engineered bacteriophages to treat drug‐resistant *Mycobacterium abscessus* was reported to show clinical improvement in a patient with cystic fibrosis (Dedrick et al., 2019). Another promising approach is the use of phage lytic proteins as antimicrobial compounds (Mondal et al., 2020), making phages a strong antibacterial contender of antibiotics.

Bacteriocins represent another category of potential antibiotic alternative. Bacteriocins are ribosomally produced antibacterial peptides produced by bacteria which themselves are immune to the killing peptide due to specific immunity mechanisms. To date, bacteriocins have been mainly used in the food industry as food safety and preservative agents (Silva et al., 2018). Bacteriocins have shown promising results as antimicrobials in animal studies. For example, mouse model studies have reported the successful use of pyocin to treat *P. aeruginosa* lung infections with high efficacy and without any adverse effects (McCaughey et al., 2016; Merrikin & Terry, 1972). Another study in mice reported that administration of nisin‐ and pediocin‐producing *Lactococcus lactis* and *P. acidilactici* strains helped reduce intestinal vancomycin‐resistant enterococci colonization (Millette et al., 2008). Bacteriocins have been successfully used to treat and prevent bovine mastitis with comparable efficacy to antibiotics (L. T. Cao et al., 2007; Crispie et al., 2004; Kitching et al., 2019). Furthermore, nisin has proven to be effective in treating mastitis caused by *Staphylococcus* in eight lactating females (Fernández et al., 2008).

Another effective way of addressing the problem of antibiotic resistance is with the use of monoclonal antibodies as alternatives or in conjunction with antibiotics. Monoclonal antibodies bypass the complications of toxicity, resistance development, and early clearance by the immune system which is seen in the case of antibiotics. The use of monoclonal antibodies for treating bacterial infections is emerging in the past few years, before which monoclonal antibodies were mostly used for treating cancer, autoimmune diseases, or viral infections (Zurawski & McLendon, 2020). A recent study in rabbits demonstrated the success of the use of monoclonal antibody obiltoxaximab against anthrax protective antigen. The authors reported that the use of obiltoxaximab improved the survival of rabbits that received a lethal dose of *B. anthracis* spores (Henning et al., 2018). In a recent clinical trial of 2655 participants, authors reported that the use of bezlotoxumab (monoclonal antibody against *C. difficile* toxin) for treating *C. difficile* infection resulted in a lower recurrence of infection (Wilcox et al., 2017). In another recent study, Watson et al. (2021) generated a monoclonal antibody from B cells of a patient to be used against *Mycobacterium tuberculosis* infection in mice. Monoclonal antibodies are costlier than producing antibiotics but have many benefits and more studies in this direction will help can help transform medicine.

One of the major advantages of bacteriocins, phages and their endolysins, and monoclonal antibodies is that they can be highly target‐specific thus rendering minimal if any, collateral damage to the microbiota.

## 9 CONCLUSION

This review has summarized the importance of the gut microbiota in host metabolism and immune functions such as immunity development, colonization resistance, cell signaling, and with the help of advanced omics technologies, the complex interactions between host and microbiota are now becoming clear. Antibiotics disrupt the microbial balance and hence the networking within the bacterial community, and that with the host. The resulting resistant bacteria make clinical treatment difficult. Due to this complex link between the host and microbiota, the current usage of antibiotics requires careful stewardship, with an emphasis on the application of antibiotic alternatives, while limiting collateral damage. To this end, we need to design, develop, and translate new antibiotic alternatives from bench to bedside, in addition to methodologies that are efficient in conserving and restoring the microbial community after antibiotic‐associated perturbations.

## CONFLICT OF INTERESTS

None declared.

## ETHICS STATEMENT

None required.

## AUTHOR CONTRIBUTIONS

**Dhrati Patangia**: Conceptualization (lead); writing – original draft (lead), writing – review and editing (equal), **Paul Ross**: Supervision (equal); conceptualization (supporting); writing – review and editing (equal), **Catherine Stanton**: Supervision (equal); conceptualization (supporting); writing – review and editing (equal), **Tony Ryan**: Supervision (equal); conceptualization (supporting); writing – review and editing (equal), **Eugene Dempsey**: Supervision (equal); conceptualization (supporting); writing – review and editing (equal).

## Data Availability

Not applicable.
